# Molecular Mechanisms in the Etiopathology of Rosacea—Systematic Review

**DOI:** 10.3390/ijms262311292

**Published:** 2025-11-22

**Authors:** Anastazja Andrusiewicz, Sofiia Khimuk, Daniel Mijas, Bohdan Shmorhun, Danuta Nowicka

**Affiliations:** 1 Faculty of Medicine, Wroclaw Medical University, Wybrzeże L. Pasteura 1, 50-367 Wrocław, Poland; 2 University Centre of General Dermatology and Oncodermatology, Department of Aesthetic Dermatology and Skin Regenerative Medicine, Faculty of Medicine, Wroclaw Medical University, ul. Borowska 213, 50-556 Wrocław, Poland

**Keywords:** rosacea, molecular mechanisms, inflammatory biomarkers, oxidative stress, toll-like receptors, NF-κB signaling

## Abstract

Rosacea is a chronic inflammatory skin disorder of multifactorial pathogenesis, in which dysregulated innate immunity, neurovascular dysfunction, oxidative stress, and microbiome imbalance are central contributors. Recent molecular studies have revealed altered cytokine expression (e.g., IL-1β, IL-6, IL-36 family), aberrant activation of signaling pathways (STAT3, NF-κB, MAPKs), and enhanced expression of innate immune receptors such as TLR2,b TLR4, and TLR7, all of which promote chronic inflammation, angiogenesis, and barrier dysfunction. This systematic review was performed according to PRISMA guidelines. A total of 1425 records were retrieved from PubMed, Scopus, and Web of Science, and 14 studies met the inclusion criteria. The included studies comprised both clinical cohorts and translational experimental investigations using human samples. Reported findings consistently confirmed systemic and tissue-specific inflammatory activity, with elevated circulating monocytes, indoleamine 2,3-dioxygenase, and inflammatory indices, as well as tissue expression of STAT3, NF-κB, MAPKs, and cathelicidin fragments. Oxidative stress markers (TOS, OSI, AOPP, MMP-9) and hypoxia-related molecules (HIF-1α) were significantly increased in patients, correlating with disease severity and vascular manifestations. Taken together, these results highlight that rosacea involves both cutaneous and systemic molecular alterations. The evidence identifies multiple biomarkers with diagnostic potential and provides mechanistic insights into immune, vascular, and metabolic dysregulation. Future research should aim to validate these findings in larger cohorts, establish standardized biomarker panels, and explore novel therapeutic strategies targeting key molecular pathways.

## 1. Introduction

Rosacea is a chronic inflammatory disorder. Its first medical description dates back to the 14th century, when the French surgeon Guy de Chauliac documented the condition [1]. The National Rosacea Society Expert Committee classifies rosacea into four subtypes: erythematotelangiectatic, papulopustular, phymatous, and ocular. Diagnosis is based on the presence of at least one primary feature in the central face, including transient or persistent erythema, papules, pustules, or telangiectasia. Secondary signs may occur simultaneously or independently and comprise burning or stinging, plaques, dry skin, edema, ocular symptoms, extrafacial involvement, and phymatous changes [2,3]. Rosacea predominantly affects the cheeks, nose, chin, and forehead, and is characterized by a chronic relapsing–remitting course with alternating phases of remission and exacerbation [4,5,6].

Epidemiological studies have demonstrated a highly variable prevalence of this condition, from below 1% to as high as 22%, influenced by differences in population characteristics, study design, and geographic or cultural factors. Onset most often occurs between the ages of 30 and 50, though cases may appear earlier or later in life. Men and women are affected with similar frequency. Fair-skinned individuals of Celtic and northern European ancestry are diagnosed more frequently, while in darker phototypes, recognition is often hindered by the less prominent visibility of erythema and telangiectasia [2,7,8].

The pathophysiology of rosacea is driven by complex interactions involving dysregulation of the immune system, neurogenic inflammation, and vascular hyperreactivity [8,9]. Although this chronic inflammatory skin disease was once regarded as a purely cutaneous disorder, accumulating evidence now associates it with a range of systemic comorbidities. Recent research suggests that individuals with the condition face an increased risk of cardiovascular disease, allergic conditions, psychiatric disorders, gastrointestinal disturbances, certain malignancies, and autoimmune diseases compared with healthy controls [10,11,12].

The pathogenesis of rosacea remains incompletely understood, but recent studies highlight its multifactorial nature with a genetic component. A variety of triggers—including ultraviolet radiation, microbial factors such as *Demodex* infestation, temperature fluctuations, certain foods, and psychological stress—are thought to initiate or exacerbate the disease [12,13].

At the molecular level, the disorder’s pathogenesis involves dysregulation of transient receptor potential (TRP) channels, which act as nonselective Ca^2+^-permeable ion channels with critical sensory and signaling functions. Their activation triggers intracellular Ca^2+^ influx, initiating downstream cascades that promote the release of vasoactive and pro-inflammatory mediators such as nitric oxide, prostaglandin I_2_, and endothelium-derived hyperpolarizing factor. Specific TRP subtypes, including TRPV1 and TRPV4, are strongly implicated in aberrant neurovascular signaling, contributing to vasodilation, inflammation, and vascular hyperreactivity observed in rosacea [14,15].

Toll-like receptors (TLRs) are implicated in inflammatory dermatoses. In rosacea, TLR2 activation increases kallikrein-5 (KLK5) from keratinocytes, generating pro-inflammatory LL-37 fragments that further stimulate mTOR signaling [16,17]. This cascade promotes chronic inflammation, although the roles of TLR2 and other TLRs remain only partly defined [18]. NF-κB signaling plays a central role in inflammatory skin diseases by regulating cytokine and chemokine production. In rosacea, excessive NF-κB activation amplifies skin inflammation. TLR7 can further enhance this pathway by recruiting MYD88, IRAK, and TRAF6, thereby driving sustained pro-inflammatory signaling [19].

Demodex antigens induce excessive production of reactive oxygen species (ROS) through activation of pro-inflammatory cytokine release and neutrophil recruitment. ROS mediate oxidative modifications of lipids and proteins, amplifying inflammatory signaling cascades and promoting endothelial dysfunction [20]. These molecular events contribute to vascular hyperreactivity and tissue injury characteristic of rosacea. Recent findings also implicate oxidative stress as a common pathogenic mechanism in both cutaneous and ocular rosacea. Biomarkers such as thiol–disulfide homeostasis serve as molecular indicators of oxidative imbalance in affected patients [21,22].

MMPs are zinc-dependent proteases involved in extracellular matrix degradation. In rosacea, MMP-2 and MMP-9 are upregulated by UV light and inflammatory cytokines, promoting collagen breakdown and tissue remodeling. Elevated MMP-9 has been detected not only in skin but also in serum and tears, suggesting both local and systemic contributions to disease pathogenesis and highlighting their value as molecular biomarkers [23].

Dendrobium polysaccharide (DOP), a glucomannan extracted from *Dendrobium candidum*, exhibits anti-inflammatory, antioxidant, and immunomodulatory properties. It has shown protective effects in various systemic disorders and skin conditions [24]. Mechanistically, DOP suppresses NF-κB activation, thereby reducing proinflammatory cytokines (TNF-α, IL-1β, IL-6), limiting oxidative stress, and preventing apoptosis through downregulation of NF-κBp65 signaling. Given the pivotal role of NF-κB in the initiation and progression of rosacea, DOP may hold therapeutic potential by modulating NF-κB–driven inflammatory and oxidative pathways [25].

Rosacea is a multifactorial inflammatory disorder whose clinical heterogeneity reflects a complex interplay of immune, vascular, neurogenic, and microbial factors. Despite significant progress in its clinical characterization, the molecular basis underlying its etiopathogenesis remains fragmented across diverse studies, limiting translation into reliable biomarkers or targeted therapies. Therefore, this review aimed to comprehensively analyze the molecular mechanisms implicated in the etiopathogenesis of rosacea. Specifically, it aimed to collate and critically analyze human studies evaluating molecular biomarkers, signaling pathways, and gene or protein expression profiles in adults with any clinical subtype of rosacea, compared with healthy or non-lesional controls. Particular emphasis was placed on innate immune dysregulation, oxidative stress, neurovascular signaling, and the interplay with microbiome-derived factors. The overarching goal was to provide an updated evidence-based molecular perspective that may bridge basic mechanistic insights with potential diagnostic and therapeutic applications.

## 2. Methods

The search was conducted on 16th March 2025, according to the Preferred Reporting Items for Systematic Reviews and Meta-Analyses (PRISMA) statement guidelines [26], using the databases PubMed, Scopus and Web of Science. The search strategy includes the following:

For PubMed: (rosacea [Title/Abstract] OR “rosacea” [MeSH Terms] OR “acne rosacea” [Title/Abstract] OR “papulopustular rosacea” [Title/Abstract] OR “erythematotelangiectatic rosacea” [Title/Abstract]) AND (“molecular mechanism” [Title/Abstract] OR “molecular mechanisms” [Title/Abstract] OR “molecular pathogenesis” [Title/Abstract] OR pathogenesis [Title/Abstract] OR “biomarkers” [MeSH Terms] OR “biomarkers” [Title/Abstract] OR “inflammatory mediators” [Title/Abstract] OR “gene expression” [Title/Abstract] OR “cytokines” [MeSH Terms] OR cytokines [Title/Abstract] OR cathelicidin [Title/Abstract] OR LL-37 [Title/Abstract] OR TLR2 [Title/Abstract] OR “innate immunity” [Title/Abstract] OR angiogenesis [Title/Abstract] OR “matrix metalloproteinases” [Title/Abstract] OR proteomics [Title/Abstract] OR transcriptomics [Title/Abstract] OR “skin microbiome” [Title/Abstract]) AND Humans [MeSH Terms] AND English [lang] AND (“1 January 2015” [Date-Publication]: “31 December 2025” [Date-Publication]) AND (“journal article” [Publication Type] NOT “review” [Publication Type])—218 documents.

For Scopus: TITLE-ABS-KEY (rosacea OR “acne rosacea” OR “papulopustular rosacea” OR “erythematotelangiectatic rosacea”) AND TITLE-ABS-KEY (“molecular mechanism” OR “molecular mechanisms” OR “molecular pathogenesis” OR pathogenesis OR biomarkers OR “inflammatory mediators” OR “gene expression” OR cytokines OR cathelicidin OR LL-37 OR TLR2 OR “innate immunity” OR angiogenesis OR “matrix metalloproteinases” OR proteomics OR transcriptomics OR “skin microbiome”) AND (PUBYEAR > 2014 AND PUBYEAR < 2026) AND (LIMIT-TO (DOCTYPE, “ar”) OR LIMIT-TO (DOCTYPE, “ar // Artykuły oryginalne”)) AND (LIMIT-TO (LANGUAGE, “English”)) AND (LIMIT-TO (SUBJAREA, “MEDI”) OR LIMIT-TO (SUBJAREA, “BIOC”) OR LIMIT-TO (SUBJAREA, “IMMU”) OR LIMIT-TO (SUBJAREA, “ENVI”))—799 documents.

For Web of Science: TS = (rosacea OR “acne rosacea” OR “papulopustular rosacea” OR “erythematotelangiectatic rosacea”) AND TS = (“molecular mechanism” OR “molecular mechanisms” OR “molecular pathogenesis” OR pathogenesis OR biomarkers OR “inflammatory mediators” OR “gene expression” OR cytokines OR cathelicidin OR LL-37 OR TLR2 OR “innate immunity” OR angiogenesis OR “matrix metalloproteinases” OR proteomics OR transcriptomics OR “skin microbiome”)—408 documents.

The records were screened independently by one reviewer at the level of title, abstract, and full text, and verified by a second reviewer. Only studies that fulfilled all predefined eligibility criteria based on the PI(E)COS framework (“Population”, “Intervention”/“Exposure”, “Comparison”, “Outcomes”, and “Study design”) [27] were included, as shown in Table 1. A comprehensive flowchart of the selection process is provided in the Results Section.

## 3. Results

### 3.1. Search Results

**Figure 1:**
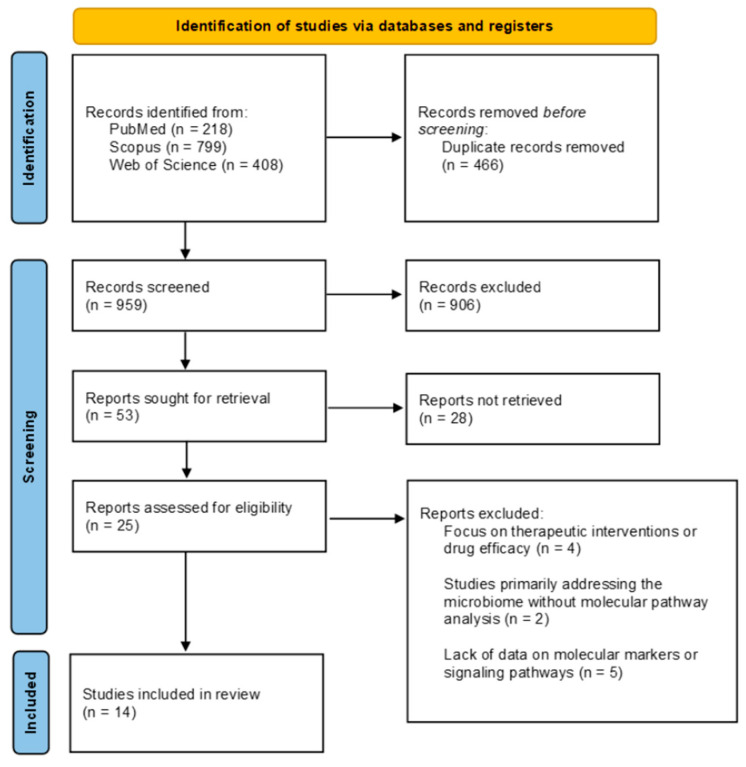
PRISMA flowchart of selected studies.

We identified 1425 records (218 in PubMed; 799 in Scopus; 408 in Web of Science). Out of these, 466 duplicate records were removed. 959 titles and abstracts were screened, and out of these, 906 were excluded. The remaining 53 articles were sought for retrieval, and 25 were assessed for eligibility. Fourteen articles met the inclusion criteria and were summarized in this review. The selection process is presented in the PRISMA flowchart (Figure 1).

### 3.2. Characteristics of Included Studies

The studies included in this review were conducted between 2017 and 2024. Research was performed in Turkey (6 studies), the USA (3), China (3), Chile (1), and France in collaboration with Turkey (1). A total of approximately 685 rosacea patients and 406 healthy controls were investigated. Among the included studies, 9 were observational case–control studies, 1 was a cohort, 3 were translational experimental studies, and 1 was a cross-sectional study. Sample sizes varied from small cohorts of 5–19 patients to larger analyses of over 100 participants, reflecting both exploratory molecular research and population-based biomarker evaluation.

The characteristics of the included studies, including study design, participants, and molecular pathways under study, are summarized in Table 2.

ARE, arylesterase activity; AOPP, advanced oxidation protein products; CCR2, C-C chemokine receptor type 2; CCL2, C-C motif chemokine ligand 2; CRP, C-reactive protein; ESR, erythrocyte sedimentation rate; HIF-1α, hypoxia-inducible factor 1-alpha; HMGB-1, high-mobility group box 1; IDO, indoleamine 2,3-dioxygenase; IL-1β, interleukin 1 beta; IL-6, interleukin 6; IL-36, interleukin 36; IL-37, interleukin 37; IL-38, interleukin 38; MAPK, mitogen-activated protein kinase; MHR, monocyte-to-high-density lipoprotein ratio; MLR, monocyte-to-lymphocyte ratio; MMP-9, matrix metalloproteinase-9; MPV, mean platelet volume; mTORC1, mechanistic target of rapamycin complex 1; NF-κB, nuclear factor kappa-light-chain-enhancer of activated B cells; NLR, neutrophil-to-lymphocyte ratio; OSI, oxidative stress index; PBMC, peripheral blood mononuclear cells; PLR, platelet-to-lymphocyte ratio; PPR, papulopustular rosacea; SII, Systemic Immune-Inflammation Index; STAT3, signal transducer and activator of transcription 3; TAS, total antioxidant status; TLR-4, toll-like receptor 4; TLR7, toll-like receptor 7; TNF-α, tumor necrosis factor alpha; TOS, total oxidant status.

**Figure 2:**
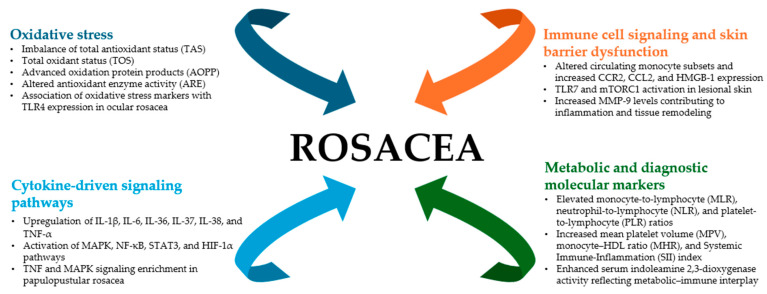
Schematic overview of molecular mechanisms in rosacea (created with PowerUser).

In analyzing the molecular mechanisms of rosacea, we categorized them into four main groups: (1) oxidative stress, (2) cytokine-driven signaling pathways, (3) immune cell signaling and skin barrier dysfunction, and (4) metabolic molecular markers. The schematic overview of these molecular mechanisms and their contribution to rosacea pathogenesis is presented in Figure 2.

### 3.3. Studies Assessing Oxidative Stress and Molecular Alterations

Of the 14 studies reviewed, 4 evaluated circulating inflammatory mediators in rosacea patients.

Gao et al. [30] examined peripheral blood mononuclear cells from 116 rosacea patients (24 males, 92 females; mean age ≈ 29.9 years), 26 systemic lupus erythematosus patients, 28 acne patients, and 42 healthy controls (20 males, 22 females; average age, mean age ≈ 30.1 years). Flow cytometry demonstrated an increased proportion of circulating classical monocytes, especially in erythematotelangiectatic rosacea. These cells expressed higher CCR2, accompanied by elevated plasma CCL2, HMGB-1, IL-1β, and TNF-α. Results showed that rosacea patients had higher frequencies of classical monocytes than healthy controls (*p* < 0.001), elevated CCR2 expression on classical and intermediate monocytes (*p* < 0.001), and increased plasma levels of HMGB 1 (*p* = 0.001), CCL2 (*p* = 0.0023), IL 1β (*p* = 0.0393), and TNF α (*p* = 0.0069), suggesting a key role of monocyte-driven inflammation in rosacea pathogenesis. Levels decreased following treatment, suggesting that monocyte-driven inflammation may be a reversible process in rosacea.

Odabasi et al. [33] assessed serum indoleamine 2,3-dioxygenase (IDO) in 52 patients with rosacea (36 females, 16 males; mean age not reported) and 29 healthy controls (21 females, 8 males; mean age not reported). Among the rosacea patients, 23 were classified as erythematotelangiectatic type and 29 as papulopustular type. Serum IDO levels were significantly higher in rosacea patients compared with controls (57.0 ± 18.1 vs. 44.5 ± 10.6 ng/mL, *p* < 0.001). Elevated levels were observed in papulopustular type (*p* = 0.001) and in both remission and exacerbation phases (*p* = 0.002). Female patients had particularly high IDO (60.0 ± 17.8 vs. 41.7 ± 9.0 ng/mL, *p* < 0.001). ROC analysis (cutoff 47.1 ng/mL) showed sensitivity of 78.8% and specificity of 60%, with stronger diagnostic value in females (83.3%/76.1%). The marker also demonstrated high diagnostic accuracy, showing strong sensitivity and specificity for distinguishing female rosacea patients from healthy individuals. These findings suggest that IDO may serve as a non-invasive biomarker reflecting systemic immune activation.

Karaosmanoglu et al. [32] evaluated systemic inflammation in 100 patients with clinically diagnosed rosacea (75 females, 25 males; mean age 49.8 ± 14.6 years) and 58 age- and sex-matched healthy controls. Rosacea patients showed higher monocyte count (*p* = 0.040), platelet count (*p* = 0.006), and MPV (*p* = 0.022) compared with controls. Inflammatory markers were also elevated: ESR (*p* = 0.006), CRP (*p* < 0.001), and SII index (*p* = 0.026). Both neutrophil-to-lymphocyte ratio (NLR) and platelet-to-lymphocyte ratio (PLR) were significantly elevated, indicating an enhanced pro-inflammatory systemic state. Authors propose NLR and PLR as cost-effective, easily obtainable markers in routine practice.

Taş-Aygar et al. [34] measured serum IL-6 and hypoxia-inducible factor-1α (HIF-1α) in 40 patients with rosacea (30 female, 10 male, aged 23–61 years, mean 40.25 ± 10.86) and 40 healthy controls (27 female, 13 male, aged 18–62 years, mean 39.7 ± 9.74). The study group comprises patients diagnosed with erythematotelangiectatic and papulopustular rosacea. Both biomarkers were elevated in patients significantly higher in rosacea patients compared to controls (*p* < 0.001 for both). HIF-1α levels were positively correlated with disease severity (r = 0.374, *p* = 0.017) and were higher in patients with telangiectasia (*p* = 0.016). No correlation was observed between IL-6 levels and disease severity (*p* = 0.844) or between IL-6 levels and the presence of telangiectasia (*p* > 0.05). This supports a mechanistic link between hypoxia-driven angiogenesis and rosacea vascular features.

### 3.4. Studies Assessing Cytokine-Driven Signaling Pathways

Of the 14 studies reviewed, four studies investigated specific cytokines and intracellular mediators in rosacea lesions.

Ekinci et al. [28] evaluated serum levels of IL-36, IL-37, and IL-38 in 50 rosacea patients (33 females, 17 males; mean age 55.4 ± 11.8 years) and 50 healthy controls (34 females, 16 males; mean age 45.4 ± 11.7 years). They observed increased IL-36 (52.2 vs. 33.0 pg/mL, *p* < 0.001) and decreased IL-37 (18.5 vs. 44.6 pg/mL, *p* < 0.001) and IL-38 (25.7 vs. 45.6 ng/L, *p* < 0.001) in rosacea patients compared with controls. No differences were detected between rosacea subtypes, and no correlations were found with age, disease onset, or duration. These findings highlight the potential of IL-36, IL-37, and IL-38 as novel molecular targets in the pathogenesis of rosacea.

Harden et al. [31] analyzed lesional and non-lesional skin explants from 5 papulopustular rosacea patients (sex not reported; mean age not reported) using transcriptomic and proteomic profiling. The study group comprises patients diagnosed with erythematotelangiectatic and papulopustular rosacea. IL-1β stimulation in vitro reproduced the inflammatory profile, implicating it as a central therapeutic target. A total of 92 differentially expressed genes (87 upregulated, 5 downregulated) and 20 proteins were identified in PPR versus NLS. IL-1β was significantly upregulated (Padj = 0.008). IL-1β stimulation of non-lesional skin induced a PPR-like profile with increased IL-6 (1.6-fold), LIF (2.4-fold), OSM (5-fold), CXCL5 (>11-fold), and CCL20 (8-fold), confirming enrichment of MAPK and TNF pathways.

Huang et al. [19] examined skin biopsies from 24 rosacea patients (female, 20–50 years) and 17 age-matched healthy female controls. The study demonstrated that TLR7 was overexpressed in rosacea lesions, activating the NF-κB/mTORC1 pathway and promoting cytokine and chemokine production in keratinocytes. Overexpression of TLR7 induced IL-1β (2.5-fold), IL-6 (3.1-fold), IL-10 (2.8-fold), and IL-12 (2.2-fold), along with chemokines CXCL2, CXCL7, and CXCL10 (2–3-fold), resulting in approximately 60% increased CD4^+^ T-cell migration. Rosacea patients also showed elevated LL-37 expression and enhanced CD4^+^ T-cell infiltration, highlighting TLR7 and LL-37 as crucial molecular triggers in disease pathogenesis.

Wang et al. [35] performed a comprehensive multi-transcriptomic analysis of skin samples from 8 rosacea patients and 6 healthy volunteers, complemented by experimental validation, complemented by analysis of public datasets (38 rosacea, 20 controls; age not reported). The study identified 719 differentially expressed genes (259 upregulated, 460 downregulated) between moderate- and high-SBD patterns, demonstrating upregulation of STAT3 signaling, TNF, IL-17, JAK/STAT, Th1/Th2 differentiation, and VEGF pathways, with increased CD4^+^ T-cell infiltration observed in patient skin. Their results demonstrate that STAT3 plays a pivotal role in linking skin barrier dysfunction to heightened inflammatory responses, suggesting that modulation of STAT3 activity may represent a potential therapeutic strategy for mitigating disease exacerbation.

### 3.5. Studies Assessing Immune Cell Signaling and Skin Barrier Dysfunction

Of the 14 studies reviewed, three studies explored receptor expression and molecular cascades related to innate immunity.

Yesilirmak et al. [38] investigated conjunctival and blood samples from 40 ocular rosacea patients (21 females, 19 males; mean age 52.64 ± 8.27 years) and 20 healthy controls (11 females, 9 males; mean age 52.90 ± 7.48 years) for TLR4 expression. The study demonstrated that TLR-4 expression was significantly higher in the conjunctival epithelium and PBMCs of patients (*p* < 0.01) and was accompanied by evidence of oxidative imbalance. Tear and serum TOS and OSI levels were significantly increased (*p* < 0.01), whereas TAS and ARE levels were significantly decreased (*p* < 0.01). Positive correlations were observed between TLR-4 and OSI, OSDI, and meiboscore (*p* < 0.05), while negative correlations were found between TLR-4 and TBUT, and between TLR-4 and Schirmer score (*p* < 0.05). These findings suggest that TLR4 activation amplifies ocular surface inflammation and barrier damage through oxidative stress mechanisms, contributing to the pathophysiology of ocular rosacea. This suggests that TLR4 activation may amplify ocular surface damage via oxidative mechanisms.

Wladis et al. [36] examined eyelid specimens from 12 rosacea patients (6 men, 6 women; mean age 57.1 ± 6.9 years) and 12 age- and sex-matched controls (6 men, 6 women; mean age 56.4 ± 7.3 years) using immunohistochemistry, and from an additional 15 rosacea patients (7 men, 8 women; mean age 57.5 ± 4.4 years) and 14 controls (7 men, 7 women; mean age 58.6 ± 4.3 years) using Western blot analysis. Both methods demonstrated enhanced NF-κB activity in rosacea eyelid tissue. Immunohistochemistry revealed a significantly higher number of phosphorylated NF-κB-positive cells in rosacea compared with controls (39.3 ± 16.9 vs. 18.4 ± 15.3 cells/40× field, *p* = 0.0024). Western blot confirmed a marked increase in the phosphorylated NF-κB/total NF-κB ratio (3.11 ± 3.53 vs. 0.58 ± 0.81, *p* = 0.0001). These analyze NF-κB activity in rosacea patients. NF-κB activation, a hallmark of chronic inflammation, supports its role as a potential therapeutic target.

Wladis et al. [37] assessed eyelid tissue from 12 rosacea patients (6 women, 6 men; mean age 57.1 ± 6.9 years) and 12 controls (6 women, 6 men; mean age 56.4 ± 7.3 years) using immunohistochemistry, and from 14 rosacea patients (8 women, 6 men; mean age 74 ± 20.4 years) and 10 controls (7 women, 3 men; mean age 74.3 ± 16.3 years) using Western blot. The study identified significantly increased phosphorylation of p38 (767.6 vs. 214.6 AU, *p* = 0.0048) and Erk1/2 (961.7 vs. 301.7 AU, *p* = 0.019) in rosacea epidermis, predominantly in keratinocytes. Western blot confirmed elevated p-p38 and p-Erk1/2 levels. Proteome array data suggested additional upregulation of STAT5a, CREB, and HSP60, indicating activation of MAPK-related signaling, although some candidate proteins were not validated by Western blot. These findings demonstrate that p38 and ERK pathway activation contribute to epidermal inflammation and remodeling, suggesting that targeted inhibition of these kinases may help preserve ocular surface barrier integrity in rosacea.

### 3.6. Studies Assessing Metabolic Molecular Markers

Of the 14 studies analyzed, three studies focused on oxidative stress parameters in rosacea.

Yesilirmak et al. [39] measured oxidative stress in serum and tears from 90 ocular rosacea patients (66 females, 24 males; mean age 51.7 ± 9.7 years) and 30 healthy controls (20 females, 10 males; mean age 48.2 ± 10.0 years). This study demonstrated the presence of oxidative stress markers (TOS, OSI) and reduced antioxidant markers (TAS, ARE) in the tears and serum of patients with ocular rosacea. In ocular rosacea patients, tear and serum TAS and ARE were significantly decreased, whereas TOS and OSI were significantly increased compared to controls (*p* < 0.01). Positive correlations were observed between tear and serum oxidative stress parameters. Tear OSI negatively correlated with TBUT and positively correlated with Meiboscore and OSDI, while no correlation was found with Schirmer score. The findings suggest that oxidative stress contributes to disease pathogenesis and may serve as an indicator of dysfunction severity.

Erdogan et al. [29] compared advanced oxidation protein products (AOPP), TAS, TOS, and OSI in 70 rosacea patients (63 females, 7 males; mean age 42.61 ± 13.02 years) and 30 healthy controls (27 females, 3 males; mean age 40.33 ± 11.50 years). Serum oxidative stress markers (TAS, TOS, AOPP) were elevated in patients with rosacea compared with controls, reflecting global redox imbalance, while OSI levels showed no difference. TAS (2.40 vs. 2.26 mmol Trolox eq/L, *p* = 0.013), TOS (3.67 vs. 2.96 μmol H_2_O_2_ eq/L, *p* = 0.034), and AOPP (101.3 vs. 63.9 μmol/L, *p* = 0.017) were significantly higher in rosacea patients compared to controls, whereas OSI levels were not significantly different (15.2 vs. 13.0, *p* = 0.151). No correlations were found between biomarker levels and disease duration, rosacea subtype, ocular involvement, symptom frequency, or family history. These results indicate systemic oxidative stress in rosacea, largely mediated by protein oxidation derived from neutrophil and monocyte activity. Fernández et al. [23] evaluated gingival crevicular fluid in rosacea patients (*n* = 18, mean age 33.05 ± 9.92 years, sex not specified) and healthy controls (*n* = 20, mean age 35.3 ± 13.98 years, sex not specified). The analysis showed that patients with rosacea had significantly higher levels of MMP-9 compared with controls (764.52 ± 569.83 pg/mL vs. 260.69 ± 170.43 pg/mL, *p* < 0.05). ROC analysis showed an AUC of 0.869 with sensitivity of 72.22% and specificity of 90.5%. Rosacea diagnosis was the main factor associated with elevated MMP-9, independent of periodontal disease, smoking, or age. The authors concluded that MMP-9 quantification in gingival crevicular fluid may serve as a reliable biomarker for rosacea, offering potential utility beyond conventional clinical assessment.

## 4. Discussion

This is the first systematic review focusing solely on the molecular mechanisms implicated in the pathogenesis of rosacea. In total, we identified that 14 studies involving human participants were included, providing an integrated overview of systemic and cutaneous biomarkers. The main conclusion is that rosacea pathophysiology is mediated by a network of interacting molecular pathways, including innate immune dysregulation, oxidative stress, vascular remodeling, and neurovascular signaling, all of which may serve as potential biomarkers or therapeutic targets.

Recent systematic reviews in the area of rosacea focus mainly on treatment [40,41,42,43,44,45], or disease burden [46,47] and the quality of life of patients with rosacea [48]. We identified only one recent systematic review dedicated to biomarkers in rosacea. This review, conducted by Geng et al. [49], included a larger number of studies; however, it also incorporated publications from non-peer-reviewed sources, which may limit the overall quality of evidence. Additionally, the Geng et al. search focused on specific biomarkers, included patients with rosacea symptoms, and included all patients regardless of age. Our review included adult patients with rosacea and focused on mechanisms rather than specific biomarkers. Nevertheless, their findings are consistent with ours, highlighting the involvement of innate immune mechanisms, particularly cathelicidin and inflammasome activation, as well as Th1- and Th17-mediated pathways. The most frequently reported biomarkers in their analysis included IL-1β, TNF-α, IL-37, IFN-γ, and MMP-9.

We analyzed evidence from studies assessing systemic inflammatory mediators. Increased circulating classical monocytes, accompanied by elevated CCR2, CCL2, HMGB-1, IL-1β, and TNF-α, point to systemic immune activation that decreases with treatment, suggesting reversibility [30]. Similarly, elevated serum IDO with high sensitivity and specificity for rosacea diagnosis highlights its value as a biomarker [33]. In parallel, inflammatory indices such as NLR and PLR also correlated with disease, supporting their role as cost-effective laboratory markers [32]. Elevated IL-6 and HIF-1α further linked hypoxia to vascular alterations, with HIF-1α correlating with telangiectasia [34].

Studies investigating cytokines and signaling molecules identified imbalances between pro- and anti-inflammatory mediators. Elevated IL-36 with reduced IL-37 and IL-38 favors sustained inflammation [28]. In papulopustular rosacea, transcriptomic–proteomic integration highlighted IL-1β as a key driver through MAPK and TNF pathways [31]. Moreover, STAT3 emerged as a hub gene linking skin barrier dysfunction to immune infiltration, emphasizing the barrier–immune axis in disease progression [35].

Innate immune receptors and downstream cascades were also implicated. TLR4 expression was increased in ocular rosacea, associated with oxidative imbalance, indicating receptor-driven amplification of oxidative stress [38]. Similarly, NF-κB signaling was elevated in eyelid tissues, consistent with its role as a central regulator of chronic inflammation [36]. MAPK pathway activation, particularly p38 and ERK, was identified in ocular tissues, linking intracellular kinase activity with local inflammation and tissue remodeling [37].

Oxidative stress was a recurrent theme. Ocular rosacea patients demonstrated increased TOS and OSI with reduced TAS, correlating with meibomian gland dysfunction severity [39]. Similarly, serum studies confirmed elevated oxidative markers, including advanced oxidation protein products [29].

Taken together, the included studies demonstrate that rosacea pathogenesis involves not only local skin changes but also systemic immune dysregulation. The findings suggest potential biomarkers, including IDO, IL-6, HIF-1α, MMP-9, and oxidative stress indices, that may be useful in diagnosis and disease monitoring. Importantly, many of these pathways converge on common effectors such as NF-κB, STAT3, and MAPK, underscoring their therapeutic relevance.

### 4.1. Limitations

Several limitations need to be acknowledged. Considerable heterogeneity was observed across studies regarding patient selection, disease subtypes, biomarkers assessed, and methodological approaches. Most studies were cross-sectional, limiting the ability to determine causality. Sample sizes were relatively small, and not all studies included appropriate controls. Furthermore, the majority focused on serum markers, with fewer analyzing lesional tissue or integrating multi-omic approaches. Due to this heterogeneity, we were not able to conduct a meta-analysis. Inconsistent reporting of outcomes in rosacea clinical trials hinders and likely prevents accurate data pooling and the performance of meta-analyses. This issue has been widely recognized, and Dirr et al. have proposed the Rosacea Core Domain Set for Clinical Trials and Practice: A Consensus Statement, which calls for standardized outcome measures in rosacea intervention studies [50].

### 4.2. Future Perspectives

Future studies should adopt standardized methodologies and larger, well-characterized patient cohorts. Longitudinal investigations are required to clarify causal relationships between molecular changes and clinical features. Integration of systemic and skin biomarkers, together with microbiome analysis, may provide a more comprehensive picture of disease pathophysiology. Ultimately, advancing our understanding of molecular mechanisms could lead to the identification of reliable biomarkers and targeted therapies, reshaping the management of rosacea.

## 5. Conclusions

These findings emphasize that rosacea should be considered a systemic inflammatory disorder with distinct molecular fingerprints across subtypes. Standardized validation of biomarkers, integration of systemic and cutaneous data, and molecularly guided stratification of patients are the next crucial steps. Central hubs such as STAT3, NF-κB, and oxidative pathways appear particularly promising as therapeutic targets, and their further investigation may ultimately enable the development of precise, mechanism-based interventions for rosacea.

## Figures and Tables

**Table 1:** Eligibility criteria according to PI(E)COS framework.

Parameter	Inclusion Criteria	Exclusion Criteria
Population	Human participants diagnosed with rosacea (any clinical subtype: erythematotelangiectatic, papulopustular, phymatous, ocular). Adults (≥18 years).	Participants without a confirmed diagnosis of rosacea. Studies including only pediatric populations (<18 years).
Intervention/Exposure	Assessment of molecular biomarkers, signaling pathways, or gene/protein expression related to the etiopathogenesis of rosacea.	Research focused primarily on therapeutic interventions without investigation of molecular mechanisms. Studies assessing only the skin or gut microbiome composition without molecular pathway analysis.
Comparison	Healthy controls; if unavailable, non-lesional skin or internal patient comparisons.	Studies without any valid comparator (no healthy controls, no non-lesional samples, no pre–post design, and no reference range).
Outcomes	Quantitative or qualitative data on molecular mechanisms (e.g., cytokines, oxidative stress markers, transcription factors, immune cell profiles, skin barrier proteins).	Lack of quantitative or qualitative data on molecular biomarkers, signaling pathways, or gene/protein expression relevant to rosacea pathogenesis. Studies reporting only clinical outcomes without molecular analysis.
Study design	Original research articles (case–control, cross-sectional, cohort, experimental studies). Studies providing data from human samples, even if combined with complementary in vitro or animal experiments.	Full version of the document not available Non-English language publications. Published before 2015 Literature reviews, editorials, commentaries, letters to the editor, and case reports.

**Table 2:** Characteristics of the included studies.

Author	Study Design	Rosacea Type	Participants	Molecular Mechanism Studied
Ekinci [28] 2024 Turkey	Observational case–control study	erythematotelangiectatic, papulopustular, ocular, phymatous	Rosacea patients (*n* = 50) Controls (*n* = 50)	IL-36, IL-37, IL-38
Erdogan [29] 2018 Turkey	Observational case–control study	papulopustular, erythematotelangiectatic, ocular	Rosacea patients (*n* = 70) Controls (*n* = 30)	Oxidative stress biomarkers: TAS, TOS, AOPP, OSI
Fernández [23] 2022 Chile	Observational case–control study	not reported	Rosacea patients (*n* = 18) Controls (*n* = 20)	MMP-9 as an inflammatory biomarker in rosacea
Gao [30] 2021 China	Cohort	erythematotelangiectatic, papulopustular, phymatous, erythematotelangiectatic overlapping papulopustular	Rosacea patients (*n* = 116) SLE patients (*n* = 26) Acne patients (*n* = 28) Controls (*n* = 42)	Circulating monocyte subsets, CCR2 expression, CCL2, HMGB-1, IL-1β, TNF-α
Harden [31] 2021 USA	Translational ex-perimental study	papulopustular	Rosacea patients (*n* = 5)	IL-1β as central mediator; MAPK and TNF signaling pathways upregulated in PPR
Huang [19] 2023 China	Translational experimental study	not reported	Rosacea patients (*n* = 24) Controls (*n* = 17)	TLR7, NF-κB and mTORC1
Karaosmanoglu [32] 2023 Turkey	Observational case–control study	erythematotelangiectatic, papulopustular, phymatous, ocular	Rosacea patients (*n* = 100) Controls (*n* = 58)	Monocytes, platelets, MPV, ESR, CRP, NLR, MLR, PLR, MHR, SII index
Odabasi [33] 2023 Turkey	Observational case–control study	erythematotelangiectatic and papulopustular	Rosacea patients (*n* = 52) Controls (*n* = 29)	Serum indoleamine 2,3-dioxygenase
Taş-Aygar [34] 2024 Turkey	Observational case–control study	erythematotelangiectatic and papulopustular	Rosacea patients (*n* = 40) Controls (*n* = 40)	IL-6, HIF-1α
Wang [35] 2022 China	Translational experimental study	not reported	Rosacea patients (*n* = 19) Controls (*n* = 10) additional validation in rosacea patients (*n* = 8) and Controls (*n* = 6)	STAT3
Wladis [36] 2019 USA	Observational case–control study	ocular	Immunohistochemistry: rosacea patients (*n* = 12) Controls (*n* = 12) Western blot: rosacea patients (*n* = 15) Controls (*n* = 14)	NF-κB signaling in rosacea skin
Wladis [37] 2017 USA	Observational case–control study	ocular	Immunohistochemistry: rosacea patients (*n* = 12) Controls (*n* = 12) Western blot: rosacea patients (*n* = 14) Controls (*n* = 16)	MAPK pathway activation via increased p38 and Erk1/2 phosphorylation in rosacea keratinocytes.
Yesilirmak [38] 2023 Turkey and France	Observational case–control study	ocular	Rosacea patients (*n* = 40) Controls (*n* = 20)	TLR-4 expression in conjunctival epithelium and PBMCs, and its association with oxidative stress markers (TAS, TOS, OSI, ARE) in ocular rosacea.
Yesilirmak [39] 2023 Turkey	Cross-sectional observational study	ocular	Rosacea patients (*n* = 90) Controls (*n* = 30)	Oxidative stress balance, including TAS, TOS, OSI and ARE activity in serum and tears.

## Data Availability

No new data were created or analyzed in this study. Data sharing is not applicable to this article.

## Abbreviations

The following abbreviations are used in this manuscript:

AOPP	Advanced oxidation protein products
ARE	Arylesterase activity
AU	Arbitrary units
AUC	Area under the curve
Ca2+	Calcium ion
CCL2	C-C motif chemokine ligand 2
CCR2	C-C chemokine receptor type 2
CD4+	Cluster of differentiation 4 positive T lymphocytes
CREB	cAMP response element-binding protein
CRP	C-reactive protein
CXCL2, 5, 7, 10	C-X-C motif chemokine ligand 2, 5, 7, 10
DOP	Dendrobium polysaccharide
ELISA	Enzyme-linked immunosorbent assay
ERK1/2	Extracellular signal-regulated kinase 1/2
ESR	Erythrocyte sedimentation rate
HC	Healthy controls
HDL	High-density lipoprotein
HIF-1α	Hypoxia-inducible factor 1-alpha
HMGB1	High-mobility group box 1
HSP60	Heat shock protein 60
IDO	Indoleamine 2,3-dioxygenase
IL-1β, 6, 10, 12, 17, 36, 37, 38	Interleukin 1 beta, 6, 10, 12, 17, 36, 37, 38
IRAK	Interleukin-1 receptor-associated kinase
JAK/STAT	Janus kinase/Signal transducer and activator of transcription
KLK5	Kallikrein-5
LDL	Low-density lipoprotein
LIF	Leukemia inhibitory factor
MAPK	Mitogen-activated protein kinase
MH	Monocyte-to-high-density lipoprotein ratio
MLR	Monocyte-to-lymphocyte ratio
MMP 2, 9	Matrix metalloproteinases 2, 9
MPV	Mean platelet volume
mTOR	Mammalian target of rapamycin
mTORC1	Mechanistic target of rapamycin complex 1
MYD88	Myeloid differentiation primary response 88
NF-κB	Nuclear factor kappa-light-chain-enhancer of activated B cells
NF-κBp65	NF-κB subunit p65
NLR	Neutrophil-to-lymphocyte ratio
NLS	Non-lesional skin
OSDI	Ocular Surface Disease Index
OSI	Oxidative stress index
OSM	Oncostatin M
PBMC	Peripheral blood mononuclear cells
PLR	Platelet-to-lymphocyte ratio
PPR	Papulopustular rosacea
qPCR	Quantitative polymerase chain reaction
RNA-seq	RNA sequencing
ROC	Receiver operating characteristic
ROS	Reactive oxygen species
RT-qPCR	Reverse transcription quantitative PCR
SBD	Skin barrier dysfunction
SII	Systemic Immune-Inflammation Index
SLE	Systemic lupus erythematosus
STAT3, 5a	Signal transducer and activator of transcription 3, 5a
TAS	Total antioxidant status
TBUT	Tear break-up time
Th1/Th2	T helper 1/T helper 2 differentiation
TLR	Toll-like receptor
TLR2, 4, 7	Toll-like receptor 2, 4, 7
TNF-α	Tumor necrosis factor alpha
TOS	Total oxidant status
TRAF6	TNF receptor–associated factor 6
TRP	Transient receptor potential channel
TRPV1, 4	Transient receptor potential vanilloid 1, 4
UV	Ultraviolet
VEGF	Vascular endothelial growth factor
WGCNA	Weighted gene co-expression network analysis
